# The Cough Reflex: The Janus of Respiratory Medicine

**DOI:** 10.3389/fphys.2021.684080

**Published:** 2021-06-29

**Authors:** Dominic L. Sykes, Alyn H. Morice

**Affiliations:** ^1^Hull University Teaching Hospitals NHS Trust, Hull, United Kingdom; ^2^Hull York Medical School, University of York, York, United Kingdom

**Keywords:** cough, chronic cough, aspiration [MeSH], airway reflux, reflux

## Abstract

In clinical practice, we commonly face adversity when encountering dysfunction of the cough reflex. Similar to ancient Roman deity Janus, it often presents with one of two opposing “faces”. Continual aberrant activation of the cough reflex, also known as chronic cough, can cause great detriment to quality of life and many of these patients are left misdiagnosed and undertreated. In contrast, loss of normal functioning of the cough reflex is the cause of a significant proportion of mortality in the elderly, primarily through the development of aspiration pneumonia. In this review we discuss both hyper- and hypo-activation of the cough reflex and how airway reflux and chronic aspiration may be involved in the aetiology and sequalae of both disease states. We detail the physiological and pharmacological mechanisms involved in cough, and how the recent development of P2X3 receptor antagonists may lead to the first pharmaceutical agent licensed for chronic cough. The treatment and prevention of loss of the cough reflex, which has been largely neglected, is also discussed as novel low-cost interventions could help prevent a number of hospital and domiciliary deaths from both acute and chronic aspiration.

## Introduction

The cough reflex is a vital defense mechanism that allows the body to expel inhaled foreign objects, potential pathogens, and endogenous secretions. Dysfunction of this reflex may be crudely dichotomized into hypo and hyper-activation. Impairment of the cough reflex is associated with aspiration and is a common mode of death in elderly, demented, and neurologically impaired patients. On the other hand, excessive chronic cough causes a major decrement in quality of life and is responsible for a large proportion of health care visits.

Chronic Cough (CC) remains one of the most frequent and onerous causes of patient morbidity encountered in healthcare and is estimated to burden 9–12% of the population (Morice, 2008; Song et al., 2015). A more worrying statistic is that despite multiple visits to healthcare professionals, only half of patients receive a diagnosis to explain their debilitating symptoms (Chamberlain et al., 2015). Several drugs demonstrate significant antitussive effect for CC such as low-dose morphine sulfate (Morice et al., 2007) and gabapentin (Ryan et al., 2012), however, many patients do not benefit from such interventions, and these centrally acting drugs have large side-effect profiles. A characteristic symptom complex, consisting of hypersensitivity to external stimuli, has been detailed recently with the new paradigm of Cough Hypersensitivity Syndrome (CHS) (Morice, 2010). In this review, we aim to analyze the current evidence for the proposed mechanisms which attempt to explain the etiology of CHS. We attempt to convey the novel paradigm of airway reflux and how it should be differentiated from traditional gastro-esophageal reflux as the culprit of cough hypersensitivity through sensitization of vagal afferents.

We also explore the second “face” of cough reflex dysfunction by detailing the sequalae of cough “hypo-sensitivity.” The harmful effects of aspiration secondary to dysfunction of the cough reflex will be examined, specifically chronic aspiration and how loss of the neural mechanisms underpinning the cough reflex is the culprit of much morbidity in the elderly.

## Chronic Cough: Establishing an Accurate Definition

Chronic cough has been defined by both the European Respiratory Society (ERS) and the American College of Chest Physicians (ACCP) as a persistent daily cough for > 8 weeks which should be based on a global clinical assessment of the patient’s symptoms and how they may relate to the many phenotypes of CC (Irwin et al., 2006; Morice et al., 2020). For the purposes of drug trials, patients with chronic cough have been stratified into one of two categories: Unexplained Chronic Cough (UCC), usually defined as cough with no obvious cause for > 1 years duration, and Refractory Chronic Cough (RCC), that being an unexplained chronic cough that persists despite rigorous investigation and treatment in accordance with best-practice guidelines (Gibson et al., 2016; Dicpinigaitis, 2020). We suggest that these definitions may be further simplified, with RCC being the failure of the treatment to improve a patient’s cough and UCC being the failure of the physician to identify the cause of the patient’s cough. These definitions have been shown to have little utility in the differentiation of patients with CC, as conveyed in COUGH-1 and COUGH-2 trials, where both UCC and RCC displayed similar symptom profiles (Morice et al., 2021). For the purposes of simplicity in this review, we will use the definition of CC as defined by the ERS and ACCP.

## Acid Reflux and Cough

Gastro-esophageal reflux disease (GORD/GERD) is caused by the retrograde transit of gastric contents into the esophagus causing esophagitis, dyspeptic symptoms sufficient to impair quality of life, or risk of long-term complications (Moayyedi and Talley, 2006). Historically it was believed that there was a causal relationship between GORD and chronic cough, with the acidic nature of the refluxate being directly responsible for the development of an continual cough (Fitzgerald et al., 1989; Irwin et al., 1989). One of the prominent theories linking GORD and chronic cough was that of proximal esophageal acidic reflux. However, previous work has shown no difference in the frequency of proximal reflux events, as measured by impedance, and pH of reflux between chronic cough patients and healthy controls (Decalmer et al., 2012).

There is evidence to suggest that cough may be directly initiated from esophageal irritation by acid and non-acid refluxate, which led to the concept of the “esophago-bronchial reflex” (Smith and Houghton, 2013). This concept is based on the theory that there may be crosstalk at the nucleus tractus solitarius (nTS) between the stimuli from esophageal and airway neurons converging in this area, which would allow reflux-mediated irritation of the esophagus to produce a “referred” initiation of the cough reflex. Numerous animal studies have evidenced this model (Canning and Mori, 2010; Chen et al., 2017, 2018, 2019). Other animal models show that mast cell activation may cause the esophagus to become more permeable to acid, increasing vagal nociceptive C-fiber activation which may lead to hypersensitivity and therefore esophageal dysfunction (Yu et al., 2014; Zhang et al., 2014). Clinical studies have corroborated the link between esophageal exposure to gastric acid and enhanced cough sensitivity using capsaicin challenge. One study demonstrated that HCl infusion into the esophagus of GORD patients increased capsaicin cough sensitivity when compared to healthy controls (Javorkova et al., 2008). A subsequent study demonstrated that patients with both non-acid and acid reflux, as measured by esophageal multi-channel intraluminal impedance combined with pH monitoring (MII-pH), had greater levels of capsaicin cough sensitivity when compared to healthy individuals (Qiu et al., 2011).

In more recent years the theory that GORD plays a pertinent role in the development of chronic cough has been contested, predominantly due to the failure of anti-acid therapy in the treatment of chronic cough (Chang et al., 2011; Kahrilas et al., 2013). An initial trial reported no difference in subjective cough counts between those with GORD and chronic cough who had their stomach pH neutralized secondary to anti-acid therapy and those whose pH remained acidic (Ours et al., 1999), suggesting that the acidic component of reflux has little effect on cough. A further randomized controlled trial showed no difference in cough questionnaire scores at 8 weeks between patients treated with proton pump inhibitors (PPI) and those who received a placebo (Faruqi et al., 2011). Researchers have attempted to establish the link between acid reflux and cough, however, the acidity of the refluxate may be less important in the etiology of cough. In this review we aim to assess the evidence for the distinct paradigm “airway reflux,” that is total reflux both acidic and non-acidic and both within the gastrointestinal tract and extra esophageal.

## What Is Airway Reflux?

Airway reflux is a distinct phenomenon from GORD. As discussed previously, GORD is defined as abnormal reflux of gastric contents, mainly considered acidic liquid and when non-acidic it is termed “volume reflux.” In contrast, airway reflux is believed to be mainly composed of fluid, either gaseous “mist” or a liquid, which may be weakly or non-acidic. This refluxate ascends through a patent esophagus and is aspirated into the upper airways, damaging the epithelium (Morice, 2013).

Airway reflux occurs as an everyday phenomenon. We all “taste” our food post-prandially on occasion. In healthy individuals, impedance monitoring detected reflux events in all participants in a “normal” cohort (Zerbib et al., 2005). A normal level of reflux and aspiration may be managed by innate defenses such as coughing and ciliary removal, which may deteriorate with increasing age. The paradigm of airway reflux has been used as an elegant explanation for the development of chronic cough (Morice, 2013). The association between cough and reflux may be historically underestimated as the term reflux was tethered to the peptic diagnostic criteria of acidic GORD.

The development of the Hull Airway Reflux Questionnaire (HARQ) has provided a validated tool to assess the likelihood of the presence of airway reflux (Morice et al., 2011). The questions that are included in the HARQ are based on what are believed to be common sequalae of gaseous, non-acidic reflux from the gastrointestinal tract. Components of the HARQ were inspired by Belafsky et al. (2002) and their Reflux Symptom Index (RSI), a tool used in the diagnosis of laryngopharyngeal reflux (LPR) which has also been termed “silent reflux”. Both of these terms are synonymous with airway reflux, “silent” reflux, whilst appropriate for voice change is inappropriate for the patient with chronic cough! The phenomena listed in the questionnaire were derived from interviews with patients with chronic cough, and are inextricably linked to the opening of the lower esophageal sphincter (Mittal and Balaban, 1997). This allows the fluid refluxate to interact with the upper and lower airways. This tool has been demonstrated to be highly sensitive in identifying those with chronic cough compared to normal volunteers with a sensitivity of 94% and a specificity of 95% (Johansson and Ternesten-Hasseus, 2016). In the recent rigorously defined cohort of CC patients participating in COUGH-1 and COUGH-2 the HARQ identified 95% of these patients as being above the upper limit of normal (Morice et al., 2021), confirming the questionnaire’s utility in the diagnosis of this patient population.

## The Esophagus and Cough

Reflux and aspiration are a complex phenomenon which is an indication of a disorder of the entire upper gastrointestinal tract. Esophageal dysmotility in patients with chronic cough has been displayed in multiple studies and is believed to be a major contributor to the pathophysiology of airway reflux. Patients with esophageal dysmotility fail to clear the residual bolus of swallowed material, giving rise to esophagopharyngeal reflux (Vardar et al., 2013; Almansa et al., 2015).

## What Is Cough Hypersensitivity Syndrome?

Historically, clinicians would diagnose patients with chronic cough with one of three principle categories: reactive airways (usually eosinophilic bronchitis or cough-variant asthma); rhinosinusitis (post-nasal drip); and GORD (Gibson, 2004). The lack of success in targeted therapy to these conditions (McGarvey, 2005), as well as the fact that a considerable proportion of patients do not accord to any of these categories, necessitates a review this classification. As many patients complain of hypersensitivity to external stimuli, CHS has been suggested as an umbrella term to globally encompass CC (Morice et al., 2014; Song and Morice, 2017). The introduction of the CHS has enabled us to understand cough-related disease as a hypersensitivity syndrome akin to chronic pain. Clinically, the history that a patient with CHS conveys is usually directly correlated with normally innocuous stimuli. However, hypersensitivity to external stimuli is not a universal finding in patients with chronic cough and must be differentiated from isolated airway reflux due to upper gastrointestinal pathology.

A pathophysiological basis for CHS is thought to be sensitization of vagal afferents in the upper and lower airways. Chronic airway inflammation may precipitate this sensitization, shifting the tussigen-cough response curve to the left leading to increased cough sensitivity to normally anodyne stimuli (Chung et al., 2013). There is also compelling evidence of an etiological mechanism within the central nervous system, as demonstrated by functional magnetic resonance imaging (fMRI) studies. Functional brain imaging techniques have shown significantly higher neural activity in the midbrain in patients with cough hypersensitivity compared to controls when both groups of participants were exposed to capsaicin (Ando et al., 2016). Interestingly these areas of the midbrain (nucleus cuneiformis and periaqueductal gray) are also known to display increased neural activity in patients with chronic pain (Lau and Vaughan, 2014), suggesting there may be a common pathophysiology between the two syndromes. Further to this, higher centers in the brain have been implicated in the development of cough hypersensitivity, with these higher centers having a role in suppressing the cough reflex (Mazzone et al., 2011). The failure of these inhibitory pathways lead to an aberrant cough reflex. Imaging has unveiled that there is reduced neural activation in the dorsomedial prefrontal and anterior mid-cingulate cortices in patients with cough hypersensitivity. This has been interpreted as a reduced ability to inhibit the activation of the cough reflex (Ando et al., 2016). These proposed central mechanisms are supported by the efficacy of centrally acting medications such as gabapentin and morphine sulfate in patients with chronic cough. The evidence for vagal hypersensitivity will be the main etiological mechanism discussed in this review, as the burgeoning interest in peripheral channel antagonists is increasing supported by clinical trial evidence.

## Vagal Afferents and the Cough Reflex

The cough reflex is primarily mediated by sensory nerves in the airways, which largely originate from the superior (jugular) and inferior (nodose) vagal ganglia (Canning et al., 2014). Of these vagal afferents Aδ fibers are responsible for the sensation of mechanical stimuli and rapid changes in pH, and are thought to be crucial to the immediate protection against acid or foreign body aspiration (Canning et al., 2006). It is currently believed that the afferent C fibers, which constitute the majority of vagal afferent fibers, are central to the development of cough hypersensitivity (Lee and Pisarri, 2001). *In vitro* studies have shown that these fibers are responsible for the mediation of the sensitization to endogenous inflammatory mediators (Grace et al., 2013). Despite these physiological principles, it must be recognized that our understanding of the wandering nerve in humans is far from complete. The exact neural composition of vagal afferents in the upper and lower airways requires further study (Mazzone and Undem, 2016). Nevertheless, vagal C fibers have attracted interest in the treatment of chronic cough, largely due their mechano-/thermo-/chemo-sensitive properties.

## Molecular Pharmacology of Cough

### TRPV1/TRPA1 Receptors

One of the most widely studied receptors expressed on vagal C-fibers is the Transient Receptor Potential Vanilloid 1 (TRPV1) channel, a ligand gated ion channel, which is known to react to irritants such as capsaicin in the upper airway to initiate the cough reflex (Jia and Lee, 2007). The TRPV1 channel has been shown to be upregulated in patients who develop chronic cough (Groneberg et al., 2004; Mitchell et al., 2005; Morice, 2010), although whether this is a primary or secondary phenomenon remains to be established. TRPV1 has also been shown to be sensitive to extracellular pH and H^+^ ions which stimulate the receptors directly, and increase sensitivity of the receptor to capsaicin (Caterina et al., 1997; Tominaga et al., 1998). This may explain the reduction in cough observed in those with severe acid reflux when treated with PPI. Transient Receptor Potential Cation Channel A1 (TRPA1), which has been shown to be co-expressed with TRPV1 (Story et al., 2003), was thought to be another vital contributor to the development of cough hypersensitivity. This receptor is expressed on 20–35% of all vagal neurons and be sensitive to a litany of compounds found in common foodstuffs including mustard oils, cinnamaldehyde, perfumes, smoke and allicin sulfides (Bandell et al., 2004; Jordt et al., 2004; Macpherson et al., 2005; Bessac and Jordt, 2008). For this reason it is believed that these TRPA1 receptors may play a greater role in cough secondary to the reflux of a gaseous mist containing food particles and products of digestion (Molyneux and Morice, 2011). Despite the promising research into both receptors, clinical trials have shown novel antagonists have no clinical benefit in patients with chronic cough (Belvisi et al., 2017; Morice, 2017). Although TRPV1 and TRPA1 play a large role in physiological irritant cough, they do not appear to be crucial in the development of pathological cough, suggesting multiple cough pathways in man. Only with the serendipitous finding that purinergic receptor blockade dramatically reduced chronic cough was a novel pathological pathway uncovered.

### The Role of ATP and the P2X3 Receptor

ATP is ubiquitous in the human body and is most famous for its role as the currency of energy in cellular metabolism. It also has multiple extracellular effects which can cause inflammation. It is thought that ATP is able to reach extracellular targets following release from cells in response to damage from exogenous and endogenous stimuli (Burnstock et al., 2012; Ford and Undem, 2013). One study demonstrated the mechanism in which nutritional lipid is phagocytosed by alveolar macrophages to produce lipid-laden macrophages (LLM). These LLMs generate activated macrophages which interact with other components of the innate immune system to promote the release of Damage-associated Molecular Patterns (DAMPS), also known as alarmins, which in turn can engender airway inflammation (Hayman et al., 2017). Airway inflammation will then induce the release of cytoplasmic alarmins, including ATP, which will then bind to extracellular targets to further propagate inflammation (Yang et al., 2017). The most prominent extracellular target pertaining to chronic cough is the P2X purinoceptor 3 (P2X3), a ligand gated cation channel which has been demonstrated to be expressed on vagal afferents, most commonly on nodose (inferior ganglion) vagal neurons which project C-fibers in the lower airways. Purinoreceptors are less commonly expressed the jugular (superior ganglion) neurons that are responsible for the innervation of upper airway structures (Undem et al., 2004). In the search for a link between ATP and CC, one study has shown that patients with CC demonstrate a greater response to inhaled ATP at lower concentrations, when compared with healthy controls (Fowles et al., 2017). This finding suggests that cough maybe mediated through the activation of P2X3, which has since become the main target for pharmaceutical development.

### TRPV4-ATP-P2X3 Signaling

Transient receptor potential cation channel subfamily vallinoid member 4 (TRPV4) has been recently implicated as a piece in the puzzle of ATP mediated cough. These receptors are activated by a vast array of stimuli including temperature, mechanical stress, and changes in pH (Nilius et al., 2004) and are extensively expressed in the airways where they may release ATP in response to hypo-osmotic stimuli (Jia et al., 2004). This ATP efflux, mediated largely by the large conductance ion pore pannexin 1 (Dahl, 2015), was then thought to directly activate the P2X3 receptors in the sensory neurons to initiate the cough reflex. Interestingly the activation of TRPV4 has been shown to cause prolonged stimulation of Aδ fibers and had no effect on the C fibers which, as discussed previously, are stimulated with activation of TRPV1 and TRPA1 channels (Bonvini et al., 2016). One study has demonstrated that antagonism of the P2X3 receptor significantly reduced cough in response to ATP and distilled water (a hypo-osmolar stimulus for TRPV4) whilst there was no effect on the response to capsaicin- citric acid-evoked cough (Morice et al., 2019). These findings again suggested that there are at least two distinct pathways responsible for generating the cough reflex, one irritant (TRPV1/TRPA1) and one pathological (P2X3). Unfortunately, investigation of a TRPV4 antagonist was stopped early due to a rise in cough counts when compared to placebo, suggesting other ATP release mechanisms may be important in CC (Ludbrook et al., 2019).

### P2X3 Inhibitors—A New Frontier for the Treatment of Chronic Cough

In recent years novel P2X3 receptor antagonists have shown much promise as a therapeutic option for patients with chronic cough (Dicpinigaitis et al., 2020). Initial proof of concept studies showed a reduction in cough frequency with P2X3 inhibitors almost 10 years ago, with all patients reporting side effects of taste disturbance (hypogeusia or ageusia) (Abdulqawi et al., 2015). Since this study, further developments of this and other P2X3 inhibitors have shown a significant reduction in objective cough count at 12-week follow-up (Morice A. et al., 2020; Niimi et al., 2020; Smith et al., 2020). Phase 3 randomized controlled trials COUGH-1 and COUGH-2 (Muccino et al., 2020) have demonstrated an 18% reduction in cough at 12 weeks and a near 15% reduction at 24 weeks vs. placebo in those treated with gefapixant. Despite a large placebo effect observed in the control group (McGarvey et al., 2020), its efficacy was confirmed and it is likely that gefapixant and other P2X3 inhibitors will soon be made widely available for patients attending cough clinics.

## Aspiration and Loss of the Cough Reflex in Respiratory Disease

Aspiration is defined as the misdirection of oropharyngeal or gastric contents into the lower respiratory tract (Marik and Kaplan, 2003). One of the most frequent and fatal manifestations of aspiration in humans is aspiration pneumonia, which has historically been attributed to the impairment of the swallowing reflex (dysphagia) causing macro-aspiration of food content and has been dubiously singularized in its definition as an acute, bacterial cause of pneumonia (Mandell and Niederman, 2019). The cough reflex, and its mechanism of protecting the airways, is completely ignored in this definition. Moreover, this definition is far too narrow and the sequalae of aspiration should be thought to include pneumonia, pneumonitis, and bronchitis. It is known that the appearance of consolidation of plain radiograph is indicative of pneumonia, whereas micro-aspiration may also lead to an “aspiration bronchitis” which, because we cannot visualize it on a radiograph, has been poorly studied. In clinical practice the term “aspiration pneumonia” is used as a blanket term for all of these conditions, and is almost always treated as a bacterial infection, when in a large proportion of cases there is likely no bacterial pathogen, but rather a chemical induced injury.

Nihei et al. provided theoretical evidence for an abacterial aspiration pneumonia, conveying that mice developed a chronic form of aspiration pneumonia, with similar histopathological findings to human lung tissue, after 28 days of chronic micro-aspiration devoid of micro-organisms (Nihei et al., 2015). This is mirrored in clinical practice where studies have shown that, in those diagnosed with aspiration pneumonia, sputum cultures seldom unveil a causative pathogen (Yamanda et al., 2010; Ebihara et al., 2011b). We suggest that the etiology of these pneumoniae may be due to the acute and/or chronic aspiration of gastro-esophageal contents into the airways. Furthermore, in studies which have elucidated bacterial pathogens, they are commonly bacteria from the *Proteobacteria* and *Firmicutes phyla* (El-Solh et al., 2003; Tokuyasu et al., 2009) and mainly of the Streptococci, Klebsiella, and Escherichia genera. This is strikingly similar to the dominant organisms of the gastric microbiome (Monstein et al., 2000; Bik et al., 2006; Andersson et al., 2008; Petra et al., 2017). This is suggestive of an intrinsic etiology in which many elderly patients aspirate refluxed contents from the gastrointestinal tract, leading to the development of aspiration pneumonia. This, however, is speculative, indeed there is still much we do not know about both the gut and lung microbiomes including how and to what extent they interact with each other in health and disease.

Satoru Ebihara and his colleagues have eloquently described a new paradigm, shifting away from the classical model of dysphagia as the solitary cause of aspiration pneumonia, instead suggesting that the keystone in the prevention of aspiration pneumonia is the cough reflex (Ebihara et al., 2016). They suggest that dysphagia and impairment of the cough reflex (referred to as “dystussia”) do not develop simultaneously in those at high risk of aspiration, such as those with dementia. Indeed, there is a time lag between the development of swallowing problems and the development of aspiration pneumonia which may be due to the fact the patient’s cough reflex deteriorates later in the progression of advanced dementia. Therefore patients are still able to expel any refluxed and/or aspirated material (Mitchell et al., 2009). Studies have demonstrated that the failure of the cough reflex, as measured by the reduction in the perception of the urge-to-cough, is strongly associated with aspiration pneumonia (Sekizawa et al., 1990; Yamanda et al., 2008). The effect of neurodegeneration of the higher centers controlling the urge-to-cough motivation system (Davenport, P. W., 2009) secondary to dementia may lead to dysfunction of the cough reflex and the consequent increased risk of aspiration.

Interestingly, it has been shown that this dysfunction is caused by the degradation of the sensory rather than the motor pathways of the cough reflex, which is associated with aging (Ebihara et al., 2011a; Malandraki et al., 2011). This has led to the development of novel techniques to protect against this apparent “cough hypo-sensitivity” in the elderly. ACE inhibitors (ACEi) are well known to increase cough sensitivity (Morice et al., 1987; Shim et al., 2020), and have demonstrated some efficacy in reducing the incidence of pneumonia in both the elderly and stroke patients (Okaishi et al., 1999; Caldeira et al., 2012). Rigorous inpatient oral care, including regular teeth and oral cavity cleaning after meals by nursing staff, may help reduce the incidence of aspiration pneumonia in elderly patients (van der Maarel-Wierink et al., 2013). Increasing cough sensitivity through capsaicin-mediated activation of TRPV1 channels has been suggested as a potential protective strategy, with data suggesting improved cough reflex, as well as improved esophageal motility in patients receiving capsaicin supplementation (Ebihara et al., 2005; Nakato et al., 2017). We suggest that we should integrate these strategies into clinical practice, as they are low-cost interventions that may well save the lives of those whose cough reflex has diminished as a result of their senescence.

Cough in the elderly has been accurately described as a double-sided conundrum (Won et al., 2018) and should be addressed at both ends of the spectrum with improved regulation of the cough reflex in order to prevent morbidity associated with hyper- and hyposensitivity.

## Conclusion

In this review, we have attempted to unmask both faces of the cough reflex in respiratory disease. We have used aspiration pneumonia as an obvious example of the damage caused by airway reflux and aspiration when the cough reflex has been impaired, however, there is evidence to suggest this mechanism could precipitate a plethora of respiratory disease (Morice and Dettmar(eds)., 2018). We have also presented the evidence for airway reflux as a common culprit of the development of CC and we have recounted recent development of P2X3R antagonists, such as gefapixant, which may well become the first drug to ever be licensed for the treatment of CC. Despite these developments, more research is required to reduce both morbidity and mortality associated with the two faces of the cough reflex.

## Author Contributions

Both authors listed have made a substantial, direct and intellectual contribution to the work, and approved it for publication.

## Conflict of Interest

The authors declare that the research was conducted in the absence of any commercial or financial relationships that could be construed as a potential conflict of interest.
